# Running-In Behavior and Failure Mechanism Between AgCuNi Alloy and Au-Electroplated Layer

**DOI:** 10.3390/s25010107

**Published:** 2024-12-27

**Authors:** Hongjian Wu, Ya’nan Zhang, Qingjian Jia, Hui Cao, Han Li, Ming Ma

**Affiliations:** 1 College of Agricultural Equipment Engineering, Henan University of Science and Technology, Luoyang 471023, China; 9906245@haust.edu.cn; 2 State Key Laboratory of Tribology in Advanced Equipment, Tsinghua University, Beijing 100084, China; caohui_mec@163.com (H.C.); han-li18@tsinghua.org.cn (H.L.); 3 College of Mechatronics Engineering, Henan University of Science and Technology, Luoyang 471003, China; 4 China Academy of Space Technology (Xi’an), Xi’an 710100, China; jiaqjkh@163.com

**Keywords:** friction behavior, transfer film, AgCuNi, Au-electroplated layer

## Abstract

To avoid wear and tear of the slip ring due to electrical corrosion, the slip ring needs to undergo the running-in process under atmospheric conditions without current after assembly. To address the urgent demand for long-service capability space conductive slip rings in the aerospace field, the running-in behavior and failure mechanism between the AgCuNi alloy and Au-electroplated layer are investigated using a ball-on-disc tribometer in this paper. The results show that the transfer film composed of Au plays an important role in modifying the friction during the sliding process. With the accumulation of wear debris composed of Ag on the disc, the contact material of the friction pair changed from Au and Au to Au, Ag and Au, so the surface roughness of wear tracks increased. Finally, the transfer film broke, which made the layer fail. This paper reveals the key element failure mechanism that causes transfer film failure in the running-in contact area, which is used to reveal the friction behavior and failure mechanism of slip ring friction pair materials, and provides a basis for the selection of running-in parameters during the running-in process of slip rings before power-on operation.

## 1. Introduction

With the continuous development of aerospace and electronics, an increasing number of electrical contact components are being widely used in various earth and space applications [1]. From a tribological perspective, the friction and wear of electrical contact materials is an important factor that determines the long-term stability of electrical contact devices [2]. Pure silver electrical contact materials have the highest electrical conductivity and thermal conductivity among all metals [3], and the price is the cheapest among noble metals. Therefore, silver has been widely used in the field of electrical contact materials. To overcome the low hardness, easy cold welding, and other shortcomings, most applications use silver-based alloys [4,5].

A slip ring is a device that is used for the transmission of electrical energy and signals between a fixed device and rotating device. It is mainly composed of a conductive ring and fiber brush. Therefore, the frictional characteristics of materials used to make the conductive ring and fiber brush directly affect the stability and service life of the slip ring.

Running-in is a process of achieving smooth motion through the operation of machine parts before the actual operation of the machine, and it allows the friction pair to reach a stable state of low friction and low wear rate [6]. It thus plays an important role in improving the friction characteristics of the sliding surface [7]. Therefore, to avoid slip ring wear caused by electrical corrosion, after the slip ring is assembled, it needs to be running-in without electricity in the atmosphere. When the running-in is over, the slip ring is energized and starts to work.

At present, there are many studies on electrical contact materials and their properties [8,9,10,11,12,13,14,15,16] and several studies investigate the running-in behavior [17,18,19,20,21,22,23,24]. The AgCuNi alloy is widely used in the design and application of spacecraft and corresponding parts. However, there are few studies on the tribological behavior of the AgCuNi alloy, and studies on the tribological behavior and mechanism during the running-in of slip rings are limited. In order to achieve better running-in and improve the service life and reliability of the slip ring, the friction behavior and failure mechanism of the friction pair must be explored.

In this study, for self-made electric brushes and conductive ring materials (AgCuNi alloy and Au-plated copper plate, respectively), running-in tests were conducted on AgCuNi alloy balls and Au-plated disks by changing the sliding speed, normal load, and friction duration. Scanning electron microscopy (SEM) and energy dispersive spectroscopy (EDS) were used to observe the morphology and elemental distribution of the worn surface, analyze the running in behavior of the AgCuNi alloy and Au--plated layer, and reveal the failure mechanism of the coating. We propose the optimal running-in parameters for the slip ring during the running-in process before power-on operation, so as to achieve a stable state of low friction and low wear rate for the friction pair. Our study offers technical guidance for improving the reliability and lifespan of conductive slip rings after operation.

## 2. Experimental Section

### 2.1. Materials

The brush material was AgCuNi25-1 prepared by the fusion casting method (74% Ag, 25% Cu, and 1% Ni) and the conductive ring material was H62 brass (62% Cu, 38% Zn) with Au plated on the surface. To study the friction behavior of the fiber brush and conductive ring, the ball-on-disc friction pair was used as the simplified friction pair of the slip ring to test. The ball material was with the same as that of the fiber brush, with a diameter of 12.7 mm. The disc material was the same as the conductive ring, with a substrate of H62 brass, and was plated with Au using the electroplating method. In addition, the thickness of the Au-electroplated layer was 3 μm, and the surface roughness (Sa) of the disc was 1.797 μm.

### 2.2. Tribology Tests

**Figure 1:**
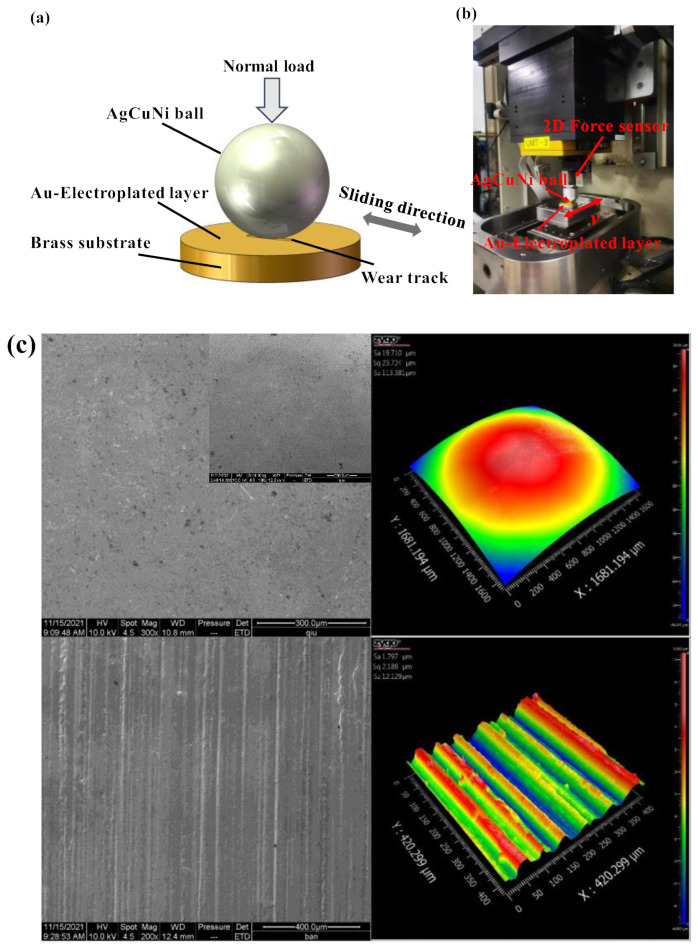
Schematic diagram and the experimental device: (**a**) schematic diagram; (**b**) experimental device; (**c**) performance of ball and Au-electroplated layer.

The tribological behaviors of the alloy ball and Au-electroplated layer were tested using a ball-on-disc reciprocating sliding tribometer (Bruker, Penang, Malaysia, Bruker Nano Inc., UMT3) (Figure 1b), and the friction behaviors of the Au-electroplated layer and alloy ball under different normal loads and sliding speeds were investigated. All tests were conducted in an atmospheric environment at room temperature of 25 °C (±1 °C). Since the maximum contact stress of the ring–brush friction pair was 325.8 MPa, when the normal load of the alloy ball was 3.0 N, the contact stress of the corresponding ball–disk friction pair for the alloy ball with a diameter of 12.7 mm was 323.6 MPa, which is close to the maximum contact stress of the ring–brush friction pair. Therefore, the normal loads applied by the alloy ball were 0.5, 1.0, 1.5, 2.0, 2.5, and 3.0 N. Similarly, the linear speed of the ring–brush friction pair was 19.9 mm/s; in order to explore the influence of the sliding speed on the friction pair, the sliding speeds of the corresponding ball–disk friction pair were 5, 10, 20, 40 and 60 mm/s, respectively. The sliding time was 60 min. In addition, the humidity was fixed at 38 ± 2%. To ensure repeatability and reliability, tribological tests were repeated at least three times.

Before the test, all test samples were cleaned with ultrasonic ethanol to remove residual dust, grease, and other solid pollutants present on their surface to keep their surface conditions as consistent as possible. During the test, the variation in the friction coefficient with time was monitored continuously. After the test, the worn surface was observed using the SEM, and the worn surfaces of the ball and disc were analyzed by EDS.

## 3. Results and Discussion

Owing to the influence of the sliding speed and normal load on the friction behavior of materials, the friction behaviors of materials at different sliding speeds were first studied, and the friction behavior was further analyzed by performing experiments under different normal loads and different sliding times.

### 3.1. Effect of Velocity and Normal Load

**Figure 2:**
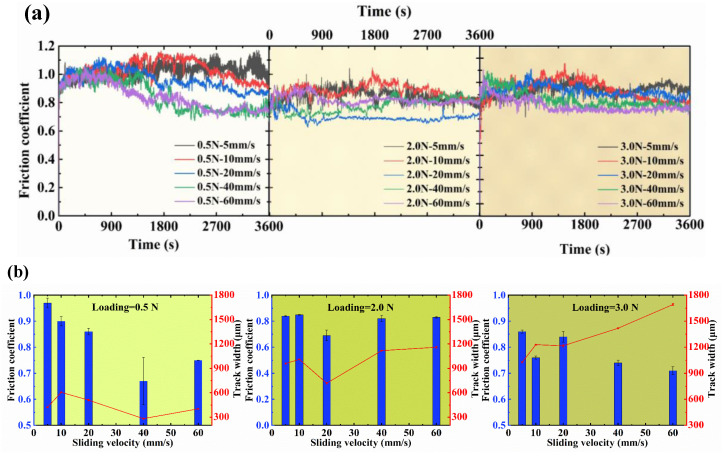

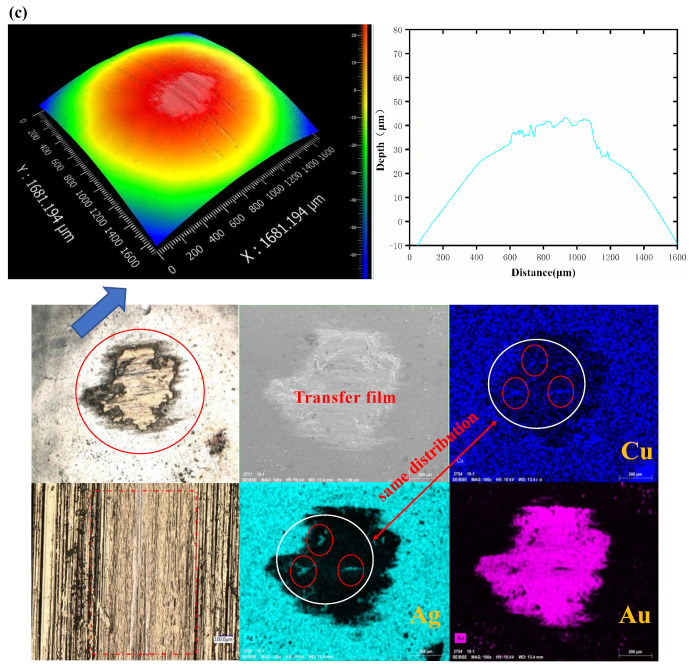

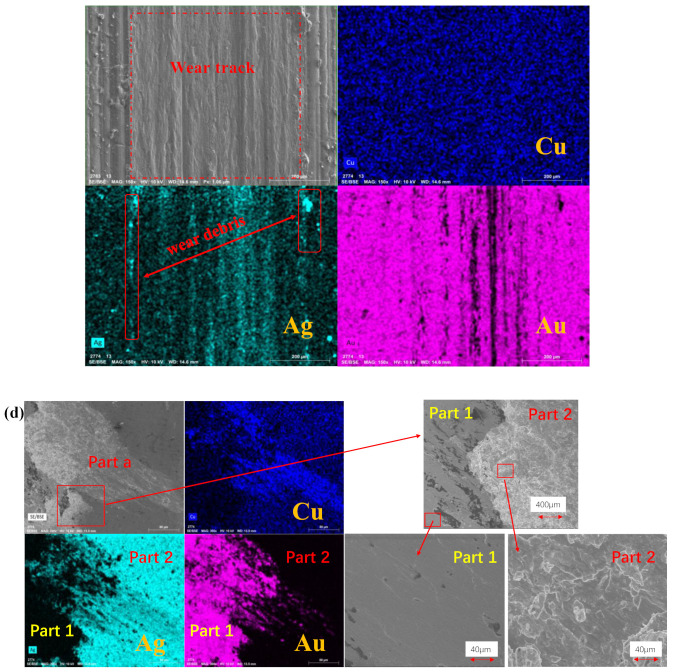

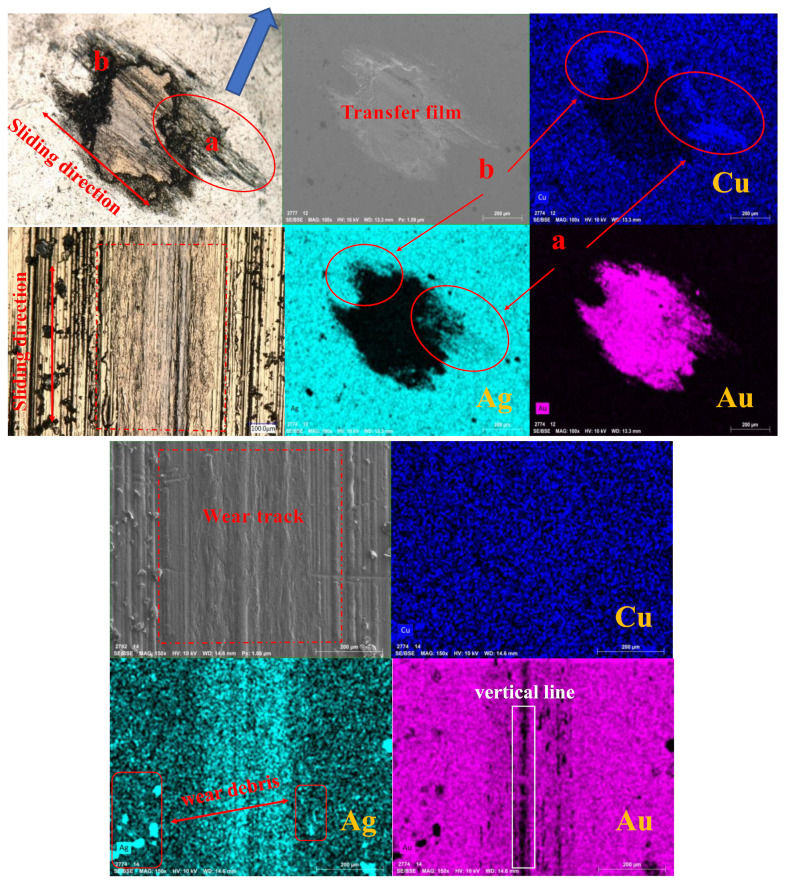
Friction coefficient, width of wear track and EDS mappings of wear scar and wear track at different sliding speeds under normal load of 0.5 N, 2.0 N and 3.0 N: (**a**) friction coefficient under different sliding speeds, (**b**) friction coefficient and wear width at different sliding speeds, (**c**) EDS mappings and 3D optical micrograph of wear scar and wear track under a normal load of 0.5 N and a sliding speed of 10 mm/s, (**d**) EDS mappings and SEM images of wear scar and wear track under a normal load of 0.5 N and a sliding speed of 60 mm/s.

Figure 2 shows the variation in the friction coefficient and width of the wear track at different sliding speeds under the normal load of 0.5 N, 2.0 N and 3.0 N.

When the normal load was 0.5 N, with the increase in the sliding speed, the friction coefficient after stabilization gradually decreased. When the sliding speed increased to 40 mm/s, the friction coefficient was a minimum of approximately 0.66. Figure 2b depicts that when the sliding speed was 40 mm/s, the wear width of the corresponding disc was the narrowest at 282.6 μm. However, when the sliding speed was 60 mm/s, the wear width increased, and the friction coefficient after stabilization was slightly higher than at a sliding speed of 40 mm/s. This is because when the sliding speed increases to 60 mm/s, the faster sliding speed accelerates the generation of friction transfer film, but the fast shear speed brings a lot of abrasive particles, so the friction coefficient during the running-in process is increased. When the friction coefficient tends to be stable, the transfer film is formed rapidly due to the high sliding speed, and a large number of abrasions accumulate and adhere to the surface of the friction film. Therefore, the friction coefficient of the friction transfer film is higher than that of the sliding speed of 40 mm/s. However, it is obviously lower than the friction coefficient when the sliding speed is 5 mm/s, 10 mm/s and 20 mm/s. This means that the sliding speed affects the production of the final friction transfer film and the antifriction effect.

To further demonstrate the effect of the sliding speed on the friction behavior when the normal load was 0.5 N, the variations in the friction coefficient and element distribution under two groups of smaller and larger sliding speed were studied. When the sliding speed was 10 mm/s, the friction coefficient increased at the initial moment, and then decreased and gradually stabilized. The width of the wear track on the disc was 605.6 μm. As shown in Figure 2c, the Au-electroplated layer on the disc underwent material transfer, and a layer of Au transfer film was formed on the surface of the alloy ball. Simultaneously, Cu and Ag remained with the same distribution on the transfer film on the alloy ball, and this part of Cu and Ag originated from the alloy ball. EDS analysis of the corresponding disc presented in Figure 2c shows that the adhesive wear of the friction pair occurred, Au from the Au-electroplated layer of the disc adhered to the surface of the alloy ball, material transfer occurred, and wear debris primarily composed of Ag formed on both sides of the wear track.

The reason for which the friction coefficient did not further decrease with the increase in sliding speed when the normal load and sliding speed were 0.5 N and 60 mm/s, respectively, was determined by observing the wear scar and wear track under these conditions, as shown in Figure 2d. It can be seen that a transfer film of Au was formed on the surface of the alloy ball. The two areas, a and b, are marked on Figure 2d and were analyzed further. The distributions of Ag and Cu in the area with no wear should be similar and uniform. However, the Cu content in parts a and b was clearly higher than other parts of the alloy ball, and the distribution of Au at part b is clearly similar to that of Cu. That is, the Cu element in parts a and b was formed by transferring the H62 brass substrate to the surface of the ball. From the distribution of Ag, Cu and Au of part a, it can be seen that there are Ag and Cu on the Au transfer film. This shows that the source of Ag on the transfer film is the same as that of Cu, i.e., this part of Ag comes from the disc. From the SEM images of part a in Figure 2d, it can be seen that the transfer film is mainly composed of two morphologies, including part 1 and part 2. Part 2 mainly consists of Ag and Cu, and part 1 mainly consists of Au. Correspondingly, from the EDS mappings of wear track, it can be seen that the wear track and debris in the disc are primarily made of Ag. And there is an apparent vertical line on the disc, primarily comprising Ag. Combined with the Au distribution of the wear track, it can be seen that the Au-electroplated layer at the vertical line has failed. In summary, according to the element analysis of the disc and alloy ball, under the conditions of a normal load of 0.5 N and a sliding speed of 60 mm/s, the Au-electroplated layer of the disc failed, owing to the contact between the alloy ball, substrate(Cu) and wear debris(Ag) and Cu and Ag from the disc were transferred to the alloy ball and were doped in the previously formed Au transfer film; thus, the friction coefficient did not decrease with the increase in sliding speed under this conditions.

To further explore the friction behavior of materials, friction tests were performed with different sliding speeds under normal loads of 2.0 N and 3.0 N (Figure 2). Under a 2.0 N normal load, Figure 2a shows that when the sliding speed was 5 mm/s and 10 mm/s, the friction coefficient first increased and then decreased, respectively. When the sliding speed was 20 mm/s, the friction coefficient first increased, and then decreased, and tended to stabilize, with a stable friction coefficient of approximately 0.69. When the sliding speed was 40 mm/s and 60 mm/s, the friction coefficient first increased and then decreased, respectively, and then increased again and tended to stabilize. Finally, it stabilized at approximately 0.82. Figure 2b shows that when the sliding speed was 20 mm/s, the width of the wear track on the corresponding disc was the narrowest (718.1 μm). Because the friction coefficient of the corresponding material was the lowest under this condition, when the sliding speed was 20 mm/s, the effect of friction reduction and anti-wear was improved. Under a 3.0 N normal load, Figure 2a shows that with the increase in the sliding speed, the friction coefficient after stabilization gradually decreased. When the sliding speed was 60 mm/s, the friction coefficient after stabilization was the lowest (approximately 0.71). However, as shown in Figure 2b, under this condition, the width of the wear track was the largest (approximately 1692.94 μm).

**Figure 3:**
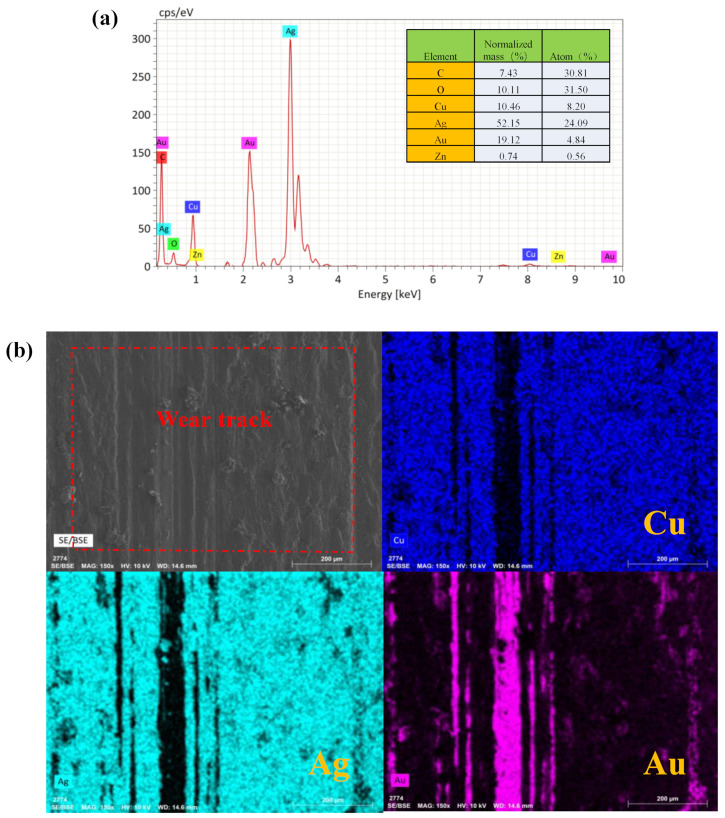

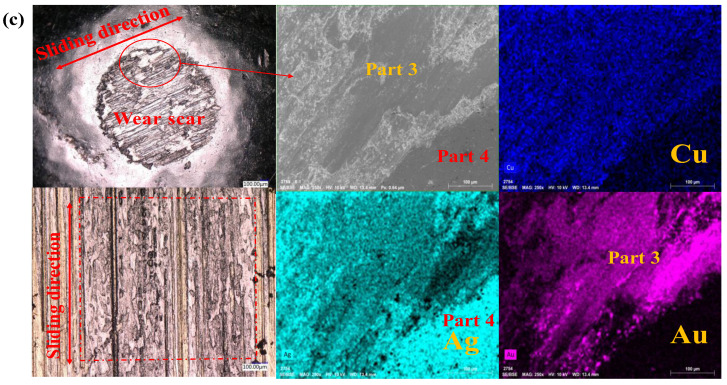
EDS mappings of wear scar and wear track under a normal load of 3.0 N and a sliding speed of 60 mm/s: (**a**) elemental analysis of the wear track, (**b**) EDS mappings of wear track, (**c**) EDS mappings of wear scar.

EDS analysis was performed on the wear scar and wear track under the condition of a sliding speed of 60 mm/s and normal load of 3.0 N. As shown in Figure 3, similar to other normal loads, the wear scar under this condition was also mainly composed of two morphologies. Additionally, part 3 was found to be dominated by Au, and part 4 was found to be dominated by Ag, respectively. From the figure of the wear scar, it can be seen that the distribution of Ag and Cu was similar, with the exception of part 4. For part 4, the distribution of Ag was found to be more than other parts, but there was almost no Cu. Therefore, the Ag in part 4 did not come from the ball itself, but from the wear debris on the wear track. As can be seen from the EDS of the wear track, the distribution of Cu and Ag was the same, but the content of Au was less and only existed in the part with a lower roughness peak without wear. Therefore, it can be said that the Au-plating layer of the disc failed under these conditions. It can be seen that the Au-electroplated layer basically disappeared and was transferred to the alloy ball. At the same time, a small amount of Zn was detected on the wear track, and this part of Zn came from the substrate H62 brass, which further indicated that the Au-electroplated layer failed and the substrate was exposed. So, the main elements on the wear track are Ag and Cu. And a large amount of Ag is also distributed on the wear scar of the alloy ball, which mainly came from the wear debris on the disc.

**Figure 4:**
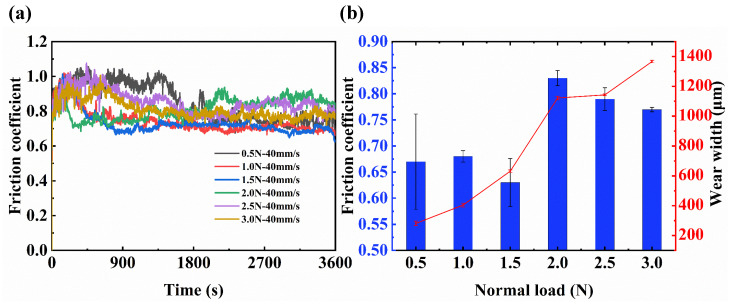

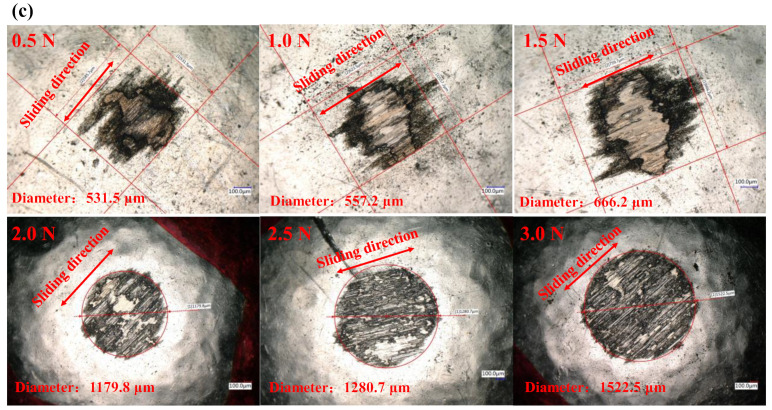
Coefficient of friction under different normal loads at a sliding speed of 40 mm/s: (**a**) friction coefficient under different normal loads; (**b**) friction coefficient and wear width with different normal loads; (**c**) wear scar surfaces under different normal loads.

In the above research, the friction coefficient and width of wear track of the friction pair were found to be the smallest when the normal load and sliding speed were 0.5 N and 40 mm/s, respectively. Therefore, to explore the influence of the normal load on the friction behavior, the friction tests for 0.5 N to 3.0 N were conducted at a sliding speed of 40 mm/s, as shown in Figure 4.

As shown in Figure 4a, when the normal load was lower than 1.5 N (0.5 N, 1.0 N, and 1.5 N), the friction coefficient first increased and then decreased, and finally tended to be stable. At the same time, as the normal load increased, the friction pair could achieve a stable friction coefficient faster. When the normal load was 2.0 N and 2.5 N, the friction coefficient first increased and then decreased, and then it again increased and finally became stable. When the normal load was 3.0 N, the variation in the friction coefficient was the same as that when the normal load was lower than 1.5 N, but the friction coefficient after stabilization was higher than that when the normal load was lower than 1.5 N. Figure 4b shows that when the normal load increased from 1.5 N to 2.0 N, the friction coefficient and wear scar diameter increased sharply, which was consistent with the classification of the variation law of friction coefficient with time. In other words, when the normal load was less than 1.5 N, the wear mechanism was inconsistent with that when the normal load was greater than 1.5 N. According to Figure 4c, when the normal load was lower than 1.5 N, a dense transfer film was formed on the surface of the alloy ball. When the normal load was 2.0 N and 2.5 N, the distribution of the transfer film on the surface of the ball was less. When the normal load was 3.0 N, the transfer film on the surface of the ball basically disappeared. It is believed that when the normal load is 0.5 N, 1 N, and 1.5 N, the Au-electroplated layer of the disc does not fail, and the transfer film is mainly composed of Au. When the normal load is 2.0 N, 2.5 N, and 3.0 N, the Au-electroplated layer of the lower sample fails, and the Ag content on Au transfer film increases gradually.

**Figure 5:**
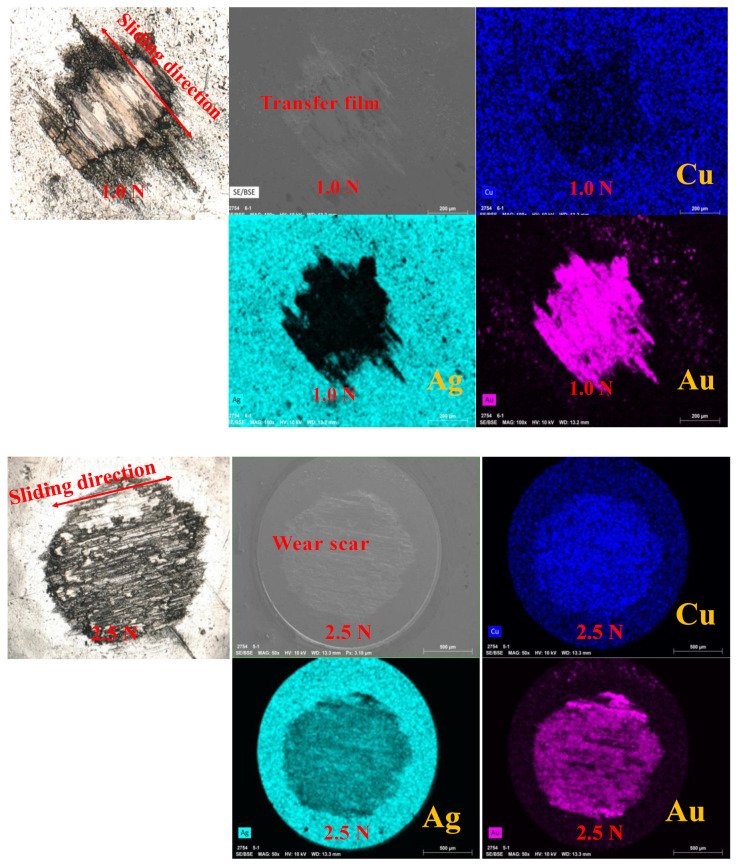
EDS mappings of wear scar under different normal loads at a sliding speed of 40 mm/s.

To verify the above results, EDS analysis was conducted on the wear surface of one of the loads of 0.5 N, 1.0 N, and 1.5 N, as well as one of the normal loads of 2.0 N, 2.5 N, and 3.0 N. As shown in Figure 5, when the normal load was 1.0 N, the Au-electroplated layer of the disc was transferred to the alloy ball, and a transfer film composed of Au was formed on the surface of the ball. When the normal load was 2.5 N, the distribution of Cu and Ag on the transfer film was completely opposite to that under the normal load of 1.0 N, Cu was mainly distributed on the transfer film, and there were also some Ag and Au on the transfer film. Therefore, the Cu on the transfer film did not originate from the ball itself, but was caused by the transfer of the disc. That is, the substrate H62 brass was in contact with the alloy ball. Thus, it can be deduced that the Au-electroplated layer exhibited failure.

**Figure 6:**
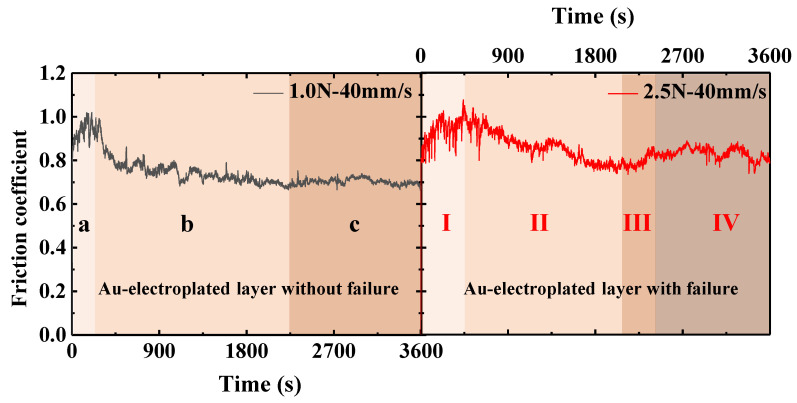
Friction coefficient curve corresponding to failure or non-failure of Au-electroplated layer: (a) high friction stage, (b) friction coefficient reduction stage, (c) friction coefficient stable stage, (I) high friction stage, (II) friction coefficient reduction stage, (III) failure stage of the Au coating, (IV) friction coefficient fluctuation stage.

Combined with the above research on the friction behavior of the AgCuNi25-1 alloy and a Au-electroplated layer under different normal loads and speeds, according to the variation in the friction coefficient with sliding time and the failure of the Au-electroplated layer, the variation in the friction coefficient with sliding time can be divided into two categories, as shown in Figure 6. Sets comprising the Au-electroplated layer with or without failure and the Au-electroplated layer with failure were taken as examples.

As shown in Figure 6, the friction coefficient of the group without failure of the Au-electroplated layer first increased with time, and then decreased, and finally tended to be stable. Therefore, the variation in the friction coefficient with sliding time can be divided into three stages. Stage a is the high friction stage, where the friction coefficient is relatively high and remains above 0.8. Stage b is the friction reduction stage, where the friction coefficient drops sharply to a lower value (less than 0.8). In Stage c, the friction coefficient maintains a stable value, which is about 0.7. Generally, the first and second stages are called the running-in process [25]. For the failure group of the Au-electroplated layer, the friction coefficient first increased and then decreased with sliding time. After a period of decrease, the friction coefficient increased again, but the final value was lower than the highest value in the initial stage, and then tended to be stable. Therefore, the change in friction coefficient with sliding time can be divided into four stages. Stage I is the high friction stage, where the friction coefficient is relatively high and remains above 0.8. Stage II is the friction coefficient reduction stage, where the friction coefficient drops sharply to a lower value (less than 0.8). Stage III is the failure stage of the Au coating. At this stage, the transfer film formed on the alloy ball is destroyed and the friction coefficient increases again. In stage IV, the contact between the alloy ball and the substrate of the Au-plated plate occurs gradually, and the friction coefficient is in a state of fluctuation.

### 3.2. Effect of Sliding Time

**Figure 7:**
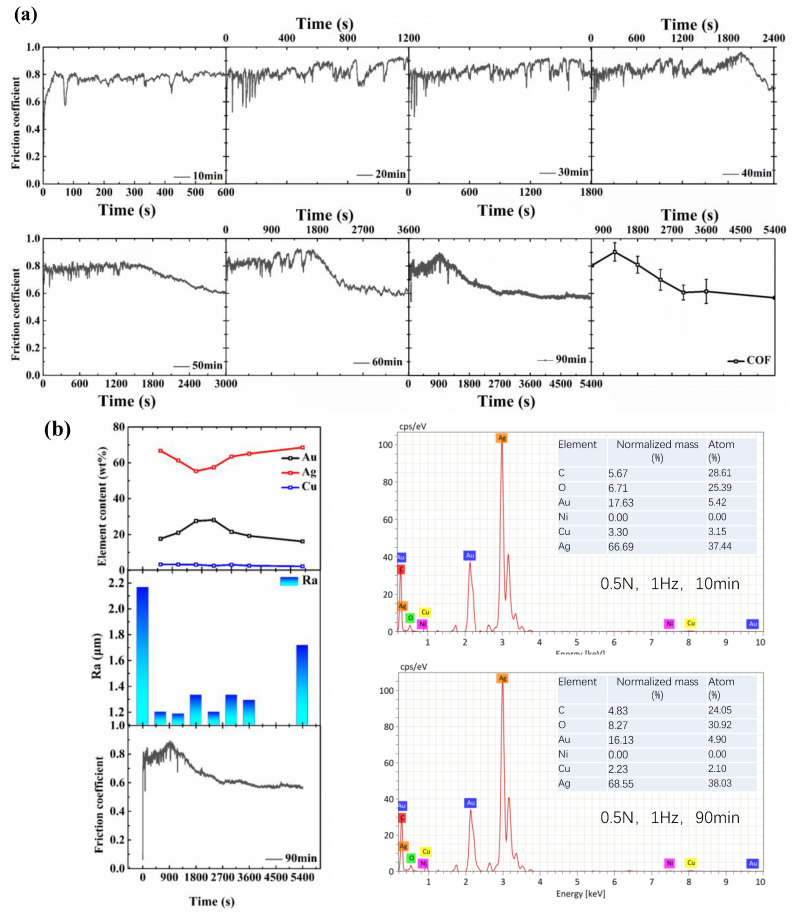

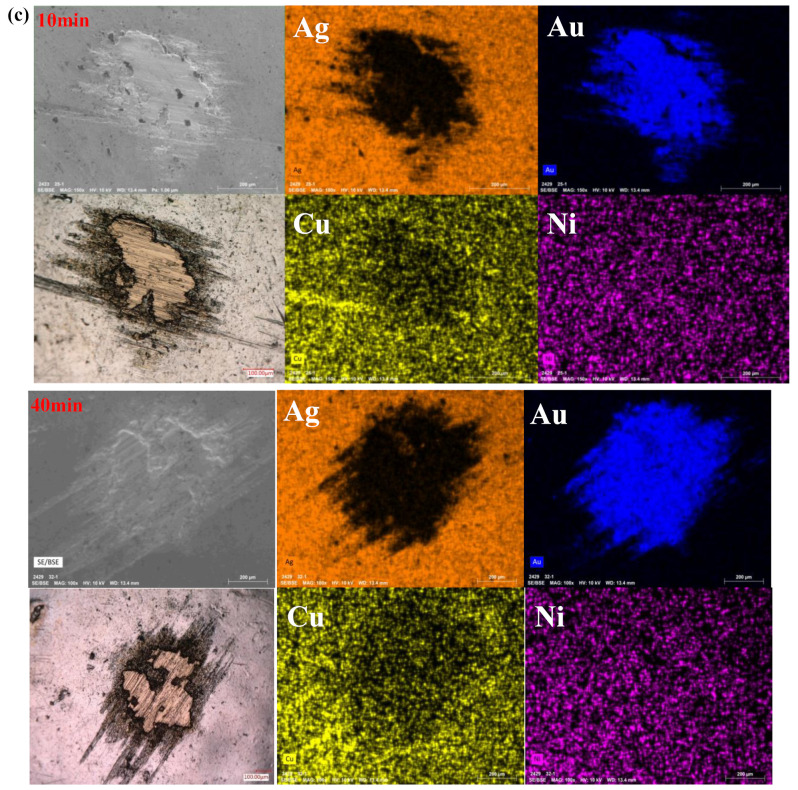

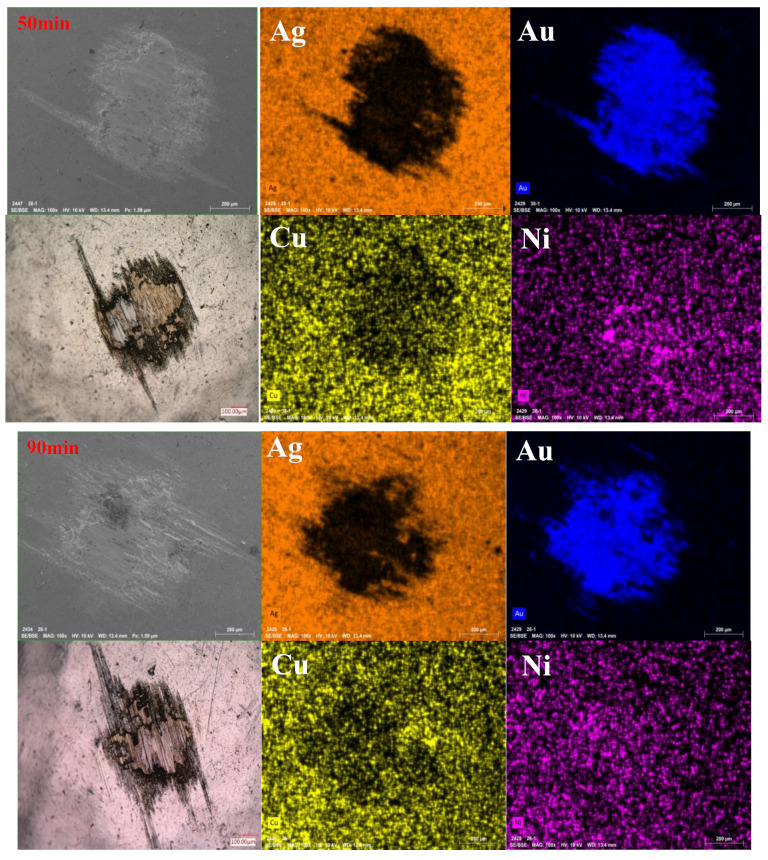

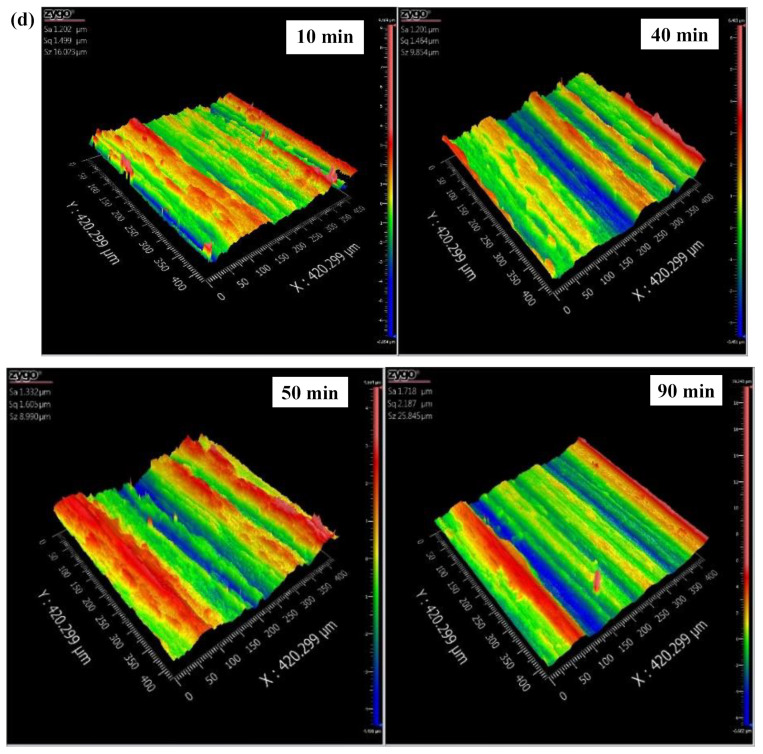
Variation in friction coefficient and element content of transfer film for different sliding times: (**a**) variation curve of friction coefficient for different values of sliding time; (**b**) element content of wear scar and roughness of wear tracks corresponding to different values of sliding time; (**c**) transfer film morphology corresponding to different sliding times; (**d**) roughness of wear tracks under different sliding times.

To further explore the friction behavior between the AgCuNi25-1 alloy and Au-electroplated layer, experiments with sliding times of 10, 20, 30, 40, 50, 60, and 90 min were conducted under a normal loading condition of 0.5 N and a sliding speed of 10 mm/s. The Au, Cu, and Ag of the transfer film that formed on the surface of the alloy ball under different sliding times were detected by the EDS. The friction coefficient and the variation in Au, Cu, and Ag content and surface roughness of wear tracks with different sliding times are shown in Figure 7.

From Figure 7a, it can be seen that when the sliding time was 90 min, the variation in the friction coefficient with time could be divided into three stages, so the Au-electroplated layer had not failed, that is, there was no stage III, which was consistent with the results of Garcia’s research on coating failure [26].

By combining the friction coefficient and change in the content of Au, Cu, and Ag with sliding time (Figure 7b), it can be seen that initially, as the friction coefficient increased with time, the content of Au also increased, which indicates the formation of the Au transfer film. As the friction coefficient gradually decreased, the Au content began to decrease slowly. As the transfer film formed, the friction coefficient tended to stabilize, and the content of the Au also tended to stabilize. Thus, the transfer film formed by Au has an important anti-friction effect for the AgCuNi25-1 alloy and the Au-electroplated layer.

The alloy ball was composed of Ag, Cu, and Ni three elements, and there was no Au element on the surface of the alloy ball before the beginning of the friction experiment. With the increase in running-in time, as shown in Figure 7b, it can be observed that initially, as the running-in time increases, the percentage of Au elements on the surface of the alloy ball increases, and the percentage of Ag elements decreases. As shown in Figure 7c, it can be observed that at the initial moment, the Au element on the Au-plated plate gradually transferred to the surface of the alloy ball to form the Au transfer film, so that the percentage of the Au element increased at this stage. At the same time, because the Au transfer film covers the alloy ball, the percentage content of Ag element decreases. As the running-in time continued to increase, the percentage of Au elements decreased, and the percentage of Ag elements increased. This is due to the accumulation of wear debris containing Ag elements adhering to the Au transfer film; therefore, the Au transfer film contains Ag elements.

**Figure 8:**
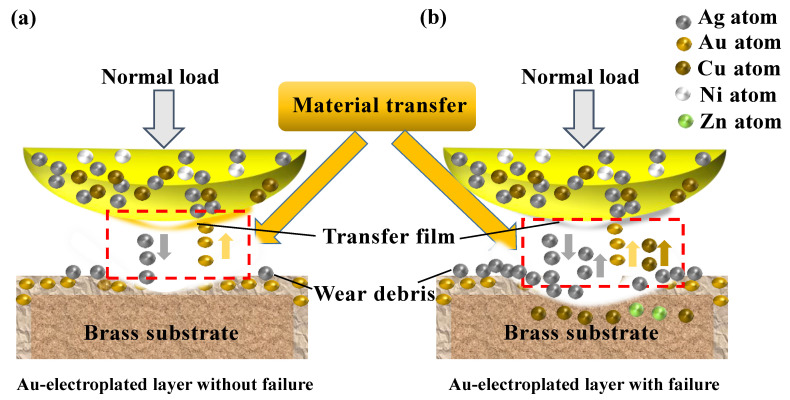
The schematics of running-in process and failure mechanism of AgCiNi25-1 alloy ball and Au-electroplated layer: (**a**) Au-electroplated layer without failure; (**b**) Failure of Au-electroplated layer.

The roughness curves of wear tracks corresponding to different sliding times are shown in Figure 7d and these show that the surface roughness of the wear track was higher initially. At the beginning of the running-in process, the alloy ball made contact with the Au-electroplated layer, and the roughness peak on the disc decreased under the cutting action of the alloy ball, so the roughness of the wear tracks decreased. As the Au from the Au-electroplated layer transferred to the alloy ball, the transfer film was formed, so the contact between the ball and the disc was the same as the contact between the transfer film (Au) and the Au-electroplated layer (Au) (Figure 8a). Owing to the mutual transfer of materials between the alloy ball and the Au-electroplated layer, the surface roughness of the wear scar fluctuated by about 1.2 μm. As the transfer film became denser, the friction coefficient tended to be stable.

As the friction time increased, with the accumulation of wear debris, the wear debris (Ag) on the Au-electroplated layer adhered to the alloy ball. So, as shown in Figure 7b, the Ag content on the Au transfer film increased gradually. Thus, when the friction coefficient tended to be stable, the contact between the ball and the plate changed from the contact between transfer film (Au) and the Au-electroplated layer (Au) during the initial period to the contact between the transfer film (Au and Ag) and the Au-electroplated layer (Au). Owing to the change in the contact material, the surface roughness of wear tracks increased after the friction coefficient became stable. With the accumulation of wear debris on the disc, the Ag on the transfer film increased gradually. Finally, the transfer film broke, the friction coefficient increased, and the wear intensified, resulting in the failure of the Au-electroplated layer and the contact between the substrate and the alloy ball (Figure 8b).

## 4. Conclusions

Based on the friction test of the electrical contact material used in the slip ring, the effects of a normal load and sliding velocity on the running-in behavior and failure mechanism between the AgCuNi alloy and Au-electroplated layer were studied. The morphology and element distribution of the worn surface were observed by performing SEM and EDS. The friction behaviors of the AgCuNi25-1 alloy and a Au-electroplated layer were analyzed, and the formation and failure mechanism of the transfer film during the running-in process were revealed. This paper proposed the optimal running-in parameters for the slip ring during the running-in process before power-on operation, so as to achieve a stable state of low friction and low wear rate for the friction pair. We also offer technical guidance for improving the reliability and lifespan of conductive slip rings after operation. The following achievements of this study are listed below:

(1) We highlighted the key element failure mechanism that causes transfer film failure in the running-in contact area. During the running-in process between the AgCuNi alloy ball and Au-electroplated layer, the transfer film (Au) formed on the alloy ball and wear debris (Ag) appeared on the Au-electroplated layer. With the accumulation of wear debris, Ag adhered to the transfer film, and the contact material of the friction pair changed from Au-Au to (Au, Ag)-Au, increasing the surface roughness of the wear tracks. Finally, with the failure of the transfer film, the friction coefficient rose, resulting in the contact between the substrate of the Au-electroplated layer and alloy ball.
(2) We revealed the rupture evolution process of the transfer film during the running-in process. According to the variation in the friction coefficient with time, the group without failure of the Au-electroplated layer could be divided into three stages. For the group with failure of the Au-electroplated layer, the friction coefficient with time could be divided into four stages. The extra stage was due to the rupture of the transfer film, which caused the friction coefficient to increase again after being reduced. The Au-electroplated layer was broken, and lost the function of reducing wear and friction.
(3) We proposed the optimal running-in parameters for the slip ring during the running-in process before power-on operation, so as to achieve a stable state of low friction and low wear rate for the friction pair. We also provided technical guidance for improving the reliability and service life of conductive slip rings after operation. During the running-in process, Au plays an important role in the anti-friction effect. Under a normal load of 0.5 N and sliding speed of 40 mm/s, the frictional and wear behaviors were the most satisfactory.

## Data Availability

All data generated or analyzed during this study are included in this published article.
